# Design and rationale of the MR-INFORM study: stress perfusion MRI to guide the management of patients with stable coronary artery disease

**DOI:** 10.1186/1532-429X-14-S1-O19

**Published:** 2012-02-01

**Authors:** Shazia T Hussain, Matthias Paul, Sven Plein, Gerry P McCann, Ajay Shah, Amedeo Chiribiri, Geraint Morton, Andreas Schuster, Mark Westwood, Divaka Perera, Michael Marber, Eike Nagel

**Affiliations:** 1 Division of imaging sciences and Biomedical engineering, kings College, London, UK; 2 Multidisciplinary Cardiovascular Research Centre, University of Leeds, Leeds, UK; 3 NIHR Leicester Cardiovascular Biomedical Research Unit, University of Leicester, Leicester, UK; 4 Cardiovascular Dept, King's College, London, UK; 5 Cardiology Dept, london Chest Hospital, London, UK

## Background

Coronary angiography and the extent of coronary luminal stenosis has historically been the main factor used in guiding decisions regarding revascularisation in patients with stable coronary artery disease. More recently, revascularisation based on invasive fractional flow reserve (FFR) measurements has been shown to result in a significant benefit for event free survival. Cardiac magnetic resonance (CMR) perfusion imaging has been shown to be superior to nuclear perfusion imaging and has the potential to become the non-invasive test of choice.

## Methods

**Figure 1:**
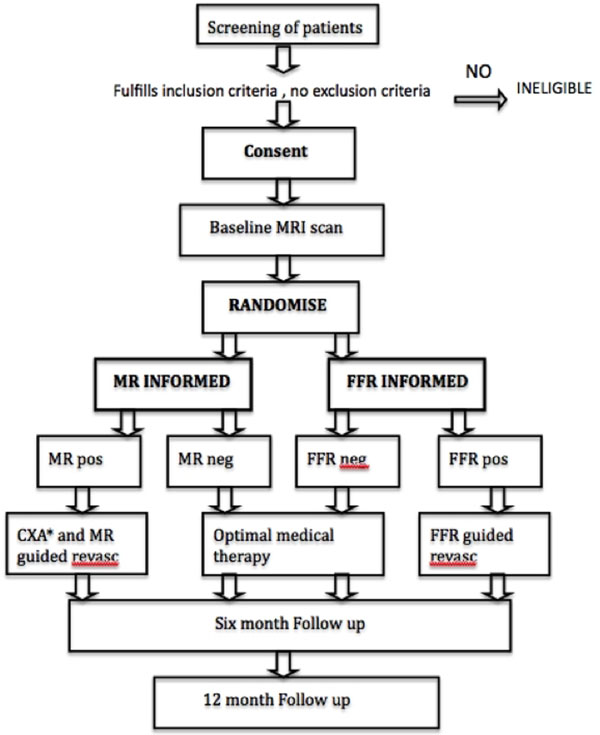
Study Flow diagram outlining the study design. *CXA = Coronary Angiography.

The MR-INFORM study is a prospective, multi-centre, randomized controlled, non-inferiority, outcome trial. It will compare the efficacy of the two investigative strategies for the management of patients with suspected coronary artery disease. Patients presenting with stable angina are randomized into two groups: The FFR-INFORMED and the MR-INFORMED group (Figure 1).

All patients undergo CMR perfusion scanning at 1.5T (various vendors) at baseline. The patients in the MR-INFORMED arm of the trial will have their subsequent management based on the results of the CMR scan. Those patients with significant ischaemia will have coronary angiography and revascularisation guided by the results of the MR scan i.e stenting in arteries that demonstrate ischaemia . All patients with non-significant ischaemia will have medical therapy optimized. The baseline scan in the FFR-INFORMED arm will remain blinded and the patient will go on to have coronary angiography with FFR measurement in those arteries with a stenosis of 40 - 99%. The revascularisation decision in this group will be based on an FFR<0.8 below which a stenosis is physiologically significant. All patients will have a cardiovascular risk assessment at baseline, 6 months and 12 months and their medical therapy is optimized.

The principal hypothesis is that selecting patients with stable angina for revascularisation and optimal medical therapy (OMT) or OMT alone based on CMR myocardial perfusion is non-inferior to selecting patients based on routine coronary angiography and fractional flow reserve (FFR) in terms of subsequent major adverse cardiac events.

The primary end-point will be the composite of major adverse cardiac events at one year, defined as death, myocardial infarction and repeat revascularisation (table 1).

**Table 1:** Detailed definition of end-points

End Point		Definition
Death		All cause mortality

Myocardial Infarction	Spontaneous	Elevation of CK or Troponin above baseline with symptoms of ischaemia, ECG changes or imaging evidence of loss of myocardium

Myocardial Infarction	Peri-procedural	CKMB >3X ULN- upper limit of normal (post PCI 12-24hrs) CKMB>5X ULN (post CABG 24-72 hours) plus new Q waves or LBBB, new native vessel or graft occlusion, imaging evidence of loss of viable myocardium

Repeat revascularisation		Repeat PCI or CABG of the target lesion performed for restenosis or other complication of the target lesion (from 5mm proximal to 5mm distal to the stent)

## Results

In recruitment phase.

## Conclusions

The MR-INFORM trial will compare the role of MR perfusion with routine coronary angiography and fractional flow measurements for guiding the management of patients with stable angina and an intermediate to high likelihood of coronary artery disease. Noninferiority of MR perfusion imaging to the current invasive reference standard (FFR) would establish MR perfusion imaging as an attractive non-invasive alternative to current diagnostic pathways. The results will help to inform national and international guidelines on the investigation and management of coronary artery disease, and ultimately lead to improved patient care.

## Funding

Biomedical Research Centre (BRC), Bayer Schering Pharma.

